# The legume-specific transcription factor E1 controls leaf morphology in soybean

**DOI:** 10.1186/s12870-021-03301-1

**Published:** 2021-11-13

**Authors:** Yongli Li, Zhihong Hou, Weiwei Li, Haiyang Li, Sijia Lu, Zhuoran Gan, Hao Du, Tai Li, Yuhang Zhang, Fanjiang Kong, Yuhan Cheng, Milan He, Lixin Ma, Chunmei Liao, Yaru Li, Lidong Dong, Baohui Liu, Qun Cheng

**Affiliations:** 1 Innovative Center of Molecular Genetics and Evolution, School of Life Sciences, Guangzhou University, Guangzhou, 516000 China; 2 College of Agriculture, Heilongjiang Bayi Agricultural University, Daqing, 163000 China; 3 Keshan Branch of Heilongjiang Academy of Agricultural Sciences, Keshan, 161606 China; 4 National Center for Soybean Improvement, National Key Laboratory of Crop Genetics and Germplasm Enhancement, Jiangsu Collaborative Innovation Center for Modern Crop Production, Nanjing Agricultural University, Nanjing, 210000 China; 5 The Innovative Academy of Seed Design, Key Laboratory of Soybean Molecular Design Breeding, Northeast Institute of Geography and Agroecology, Chinese Academy of Sciences, Harbin, 150000 China; 6 University of Chinese Academy of Sciences, Beijing, 100000 China; 7 Beijing International Urban Agricultural Science and Technology Park, Zhong Nong Fu Tong, Beijng, 100000 China

**Keywords:** Soybean, *E1*, Transcription factor, Overexpression, Transgenic plant, Leaf development

## Abstract

**Background:**

The leaf is a determinate organ essential for photosynthesis, whose size and shape determine plant architecture and strongly affect agronomic traits. In soybean, the molecular mechanism of leaf development is not well understood. The flowering repressor gene *E1*, which encodes a legume-specific B3-like protein, is known to be the gene with the largest influence on soybean flowering and maturity. However, knowledge of its potential other functions remains poor.

**Results:**

Here, we identified a novel function of E1 protein in leaf development. Unifoliolate leaves of *E1-*overexpression (*E1*-*OE*) lines were smaller and curlier than those of wild type DongNong 50 (DN50) and Williams 82 (W82). Transverse histological sections showed disorganized cells and significantly elevated palisade tissue number, spongy tissue number, and bulliform cell number in *E1-OE* lines. Our results indicate that E1 binds to the promoters of the leaf- development-related *CINCINNATA* (*CIN*)-*like TEOSINTE BRANCHED1*/*CYCLOIDEA*/*PROLIFERATING CELL FACTOR* (*TCP*) transcription factor genes to negatively regulate their expression.

**Conclusions:**

Our findings identify E1 as an important new factor in soybean leaf development.

**Supplementary Information:**

The online version contains supplementary material available at 10.1186/s12870-021-03301-1.

## Background

The shape of the leaf, the main photosynthetic organ in plants, varies based on species and developmental stage [1–3]. Leaf size and shape are important for leaf function and plant survival [4–7]. Genes that influence leaf formation have been described in rice, *Cardamine*, tomato, and *Arabidopsis thaliana* [1, 3, 8–11]. For example, *TCP3* gene regulates *Arabidopsis* leaf development via the jasmonate signaling pathway [12, 13]. In rice, *curled later1* (*cur1*) mutants have narrow leaves compared with wild type (WT) [3]. Little is known, however, about the molecular mechanisms of leaf development of in soybean (*Glycine max*), despite its status as an economically important plant oil and protein crop.

In 1927, a pair of genes controlling soybean maturity were detected and named *E* and *e* [14]. In 1971, Bernard confirmed that these genes are identical to *E1* and *e1*, two alleles of a major locus that influencing maturity [15]. The *E1* locus has a largest impact on flowering time and maturity in cultivated soybean [16–18]. Xia et al. mapped *E1* through positional cloning, and discovered that it contained a putative nuclear localization signal and sequences related to the plant-specific B3 domain [19]. The E1 protein is assumed to be a transcription factor unique to legumes [18, 19]. In cultivated soybean, it inhibits flowering: the leaky allele (*e1*-*as*) and the loss-of-function alleles (*e1*-*fs* and *e1*-*nl*) cause earlier flowering under long-day (LD) conditions [17, 19, 20]. The molecular mechanism of E1 regulation of soybean flowering has been well studied, with both genes acting upstream of *E1* (such as *E3*, *E4*, *J*, *LHYs*, *Tof11*, and *Tof12*) [19–22] and genes acting downstream of *E1* (such as *FT2a* and *FT5a*) reported [19, 23–25]. In soybean, some important flowering genes have different functions in other aspects of plant development [26, 27]. For instance, *FT5a* have dual function in the regulation of post-flowering stem growth and flowering time [27]. Nonetheless, possible roles of E1 in other developmental processes are unknown.

In Arabidopsis, microRNA *miR319A*/ *JAGGED AND WAVY* (*JAW*), a key role in leaf development, causing a wavy-leaf phenotype by suppresses CINCINNATA (CIN) subclass *TEOSINTE BRANCHED1/CYCLOIDEA/PROLIFERATING CELL FACTOR* (*TCP*) genes (*TCP2*, *TCP3*, *TCP4*, *TCP10*, and *TCP24*) [12]. The TCP proteins comprise one of the largest families of plant-specific transcription factors [28–30]. Among them, CINCINNATA (CIN) subclass *TCP* genes contribute to regulate the leaf development [10, 12, 31–33]. The soybean genome encodes 54 TCP transcription factors that fall into two classes: 26 members in class I group TCPs and 28 in class II group TCPs. Class II TCP members are further divided into the CIN subclass (19 TCPs) and the CYCLOIDEA/ TEOSINTE BRANCHED1 (CYC/TB1) subclass (9 TCPs) [30]. In this study, we uncovered a role of *E1* in soybean leaf development, finding that E1 directly represses CIN-type *TCP* genes (*TCP14*, *TCP29*), resulting in small, curly unifoliolate leaves.

## Results

### Overexpression of *E1* influences leaf development

**Fig. 1:**
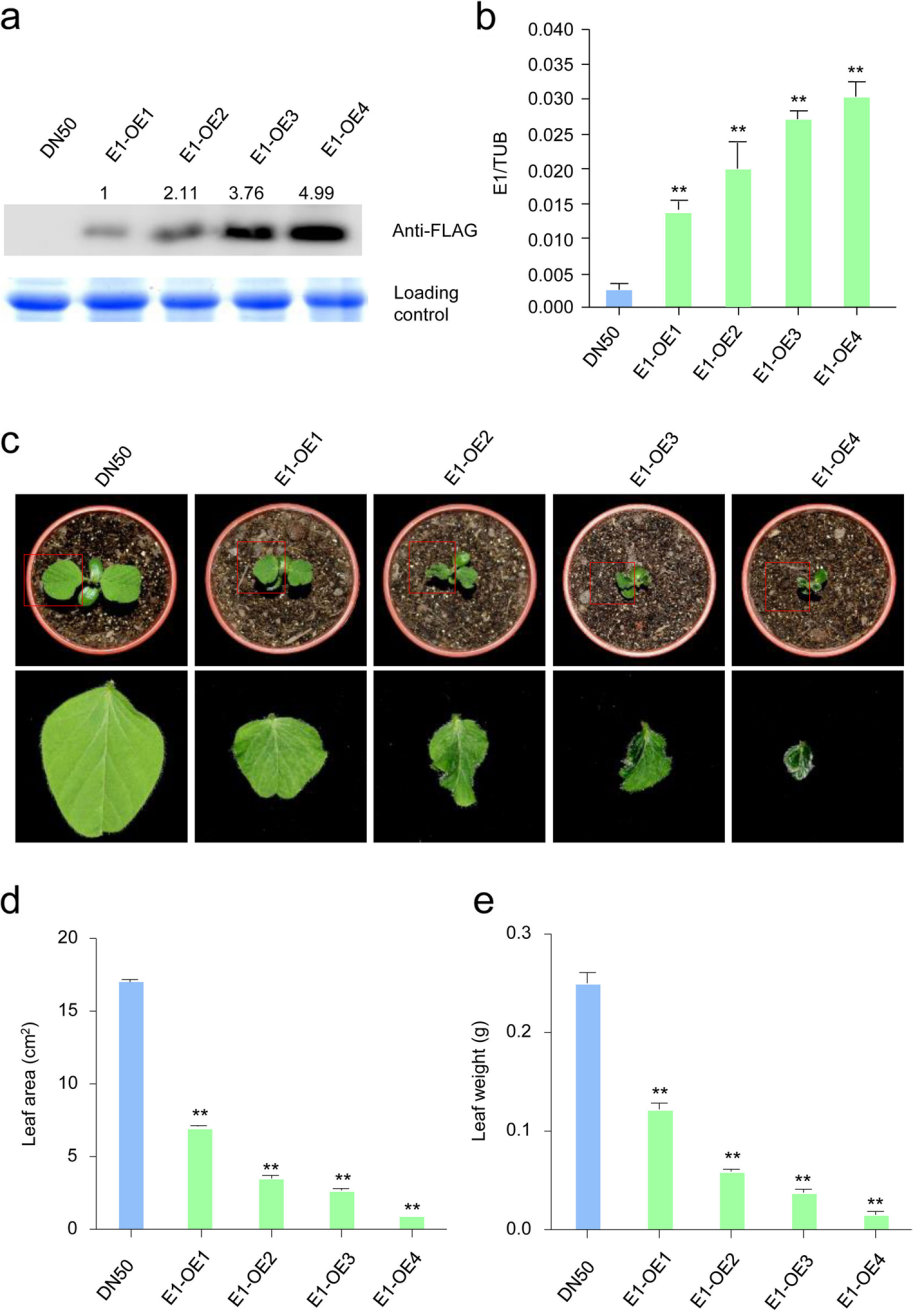
The leaf phenotypic characterization of the *E1-overexpression* (*E1*-*OE*) plants in DN50. **a**. Immuno-blot of FLAG antibodies in DN50 and *E1-OE* lines. The gel image had high-contrast and the original gel image included in the Additional file 2. **b**. The expression of *E1* in DN50 and *E1-OE* plants. The soybean *TUB* (*GmTubulin*) gene was used as an internal control to normalize gene expression data. The experiment was performed using three biological replicates, each with three technical replicates. Differences between groups were statistically analyzed using Student’s *t*-test (**P < 0.01). Bars indicate standard error of the mean. **c**. Top view of DN50 and *E1-OE* plants. Red boxes indicates that the close-up image of the leaf of DN50 and *E1-OE* plants. **d**. Quantification of leaf size in DN50 and *E1-OE* plants (*n* = 10). **e**. Quantification of leaf weight in DN50 and *E1-OE* plants (n = 10). All values are presented as mean ± standard error of the mean (s.e.m.) (n = 10 plants). Bars indicate the s.e.m. Significant differences were identified by Student’s t-test (**P < 0.01)

To examine the function of *E1* (*Glyma.06G207800*) in other developmental pathways, we created four independent transgenic lines that express *p35S:E1-Flag* in DN50. Immunoblot analyses confirmed the expression of the recombinant E1 fusion protein in four independent T_7_ *E1-OE* lines (*E1*-*OE1*, *E1*-*OE2*, *E1*-*OE3*, and *E1*-*OE4*). The expression of the E1 protein line was highest in *E1*-*OE4*, followed by *E1*-*OE3*, *E1*-*OE2*, and *E1*-*OE1* (Fig. 1a). Quantitative reverse transcription-PCR (qRT-PCR) analyses confirmed that the expression level of *E1* was significantly higher in *E1*-*OE* lines than in DN50, and that *E1* was constitutively and highly expressed in the *E1*-*OE4* line, followed by *E1*-*OE3*, *E1*-*OE2*, and *E1*-*OE1* (Fig. 1b).

As compared with DN50, *E1*-*OE* transgenic plants flowered significantly later under long-day conditions, and the plants were much shorter (Additional file 1: Fig. S1a-b). Assessment of the unifoliolate leaves of *E1*-*OE* and DN50 plants at 7 DAE, revealed that *E1*-*OE* plants had smaller leaf areas and lower leaf weights than DN50 (Fig. 1c-e), they also curled downward (Fig. 1c). Higher *E1* expression in the *E1*-*OE* lines was associated with increased curliness of the leaves (Fig. 1c).

We also observed the phenotypes of T_1_ *E1*-*OE* transgenic lines in the Williams82 (W82) background. Consistent with our observations of *E1*-*OE* transgenics in DN50, leaf area and weight of *E1-OE* plants (*E1*-*OE5*–*8*) were smaller and lighter than those of W82 (Additional file 1: Fig. S2a-c). Thus, *E1* may regulate leaf development in soybean. Since the genetic stability of T_7_ *E1-OE* lines in DN50 was higher than that of T1 *E1-OE* lines in W82, the *E1*-*OE* transgenic lines in DN50 were selected for subsequent experiments.

### E1 regulates cell number and size in the developing leaf

**Fig. 2:**
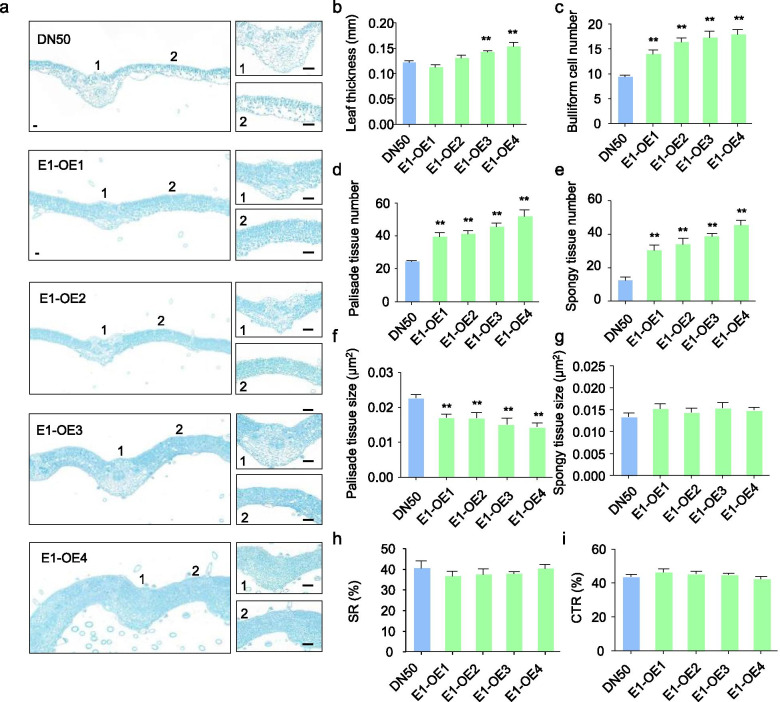
The cell size and cell number of the leaf in DN50 and *E1-OE* plants. **a**. Transverse sections of leaves from DN50 and *E1-OE* lines. **b**. Quantification of leaf thickness. **c**. Bulliform cell number. **d**. Palisade tissue number. **e**. Spongy tissue number. **f**. Palisade tissue size. **g**. Spongy tissue size. **h**. spongy tissue ratio. **i**. cell tense ratio

Proper balance of leaf tissue structure is critical for normal leaf development [34]. To further our understanding of the processes controlling leaf development, we analyzed transverse histological sections of *E1-OE* unifoliolate leaves. Compared with those in DN50 plants, cells were more disorganized in *E1-OE* plants (Fig. 2a). As an additional approach to examine the effects of *E1-OE*, we compared leaf functional traits such as leaf thickness, the cell tense ratio (CTR), spongy tissue ratio (SR), cell number and cell size (Fig. 2b-i). Leaf thickness was similar in *E1*-*OE1* and *E1*-*OE2* lines, but increased in *E1*-*OE3* and *E1*-*OE4* lines (Fig. 2b). The CTR, SR, and spongy tissue size were similar in *E1-OE* and DN50 plants (Fig. 2g, h, i). In contrast, *E1-OE* plants had significantly higher palisade tissue number, spongy tissue number and bulliform cell number (Fig. 2c-e), and lower palisade tissue size (Fig. 2f), confirming that the *E1* could regulate leaf development by affecting the leaf tissue structure. We found that higher expression of *E1* was associated with a more obvious cellular phenotype, confirming that E1 could influence the balance of different cells within the leaf tissue.

### RNA-seq analysis of *E1* overexpression

**Fig. 3:**
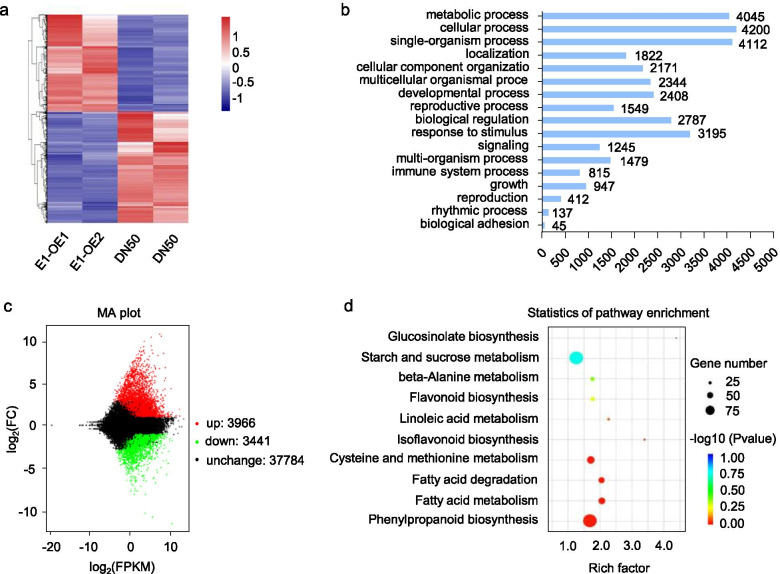
Differentially expressed genes identified from RNA-seq analysis. **a**. The heat map of differentially expressed genes in DN50 and *E1-OE* lines. The numerical values for the blue-to-red gradient bar represent log2-fold change relative to the control sample. **b**. GO terms that were statistically enriched in differentially expressed genes in DN50 and *E1-OE* lines according to the RNA-seq assay. **c**. The numbers of genes showing differential expression between DN50 and *E1-OE* plants. **d**. KEGG pathway that were statistically enriched in DN50 and *E1-OE* RNA-seq assay. The dot size indicates the number of DEGs of the pathway, and the red-to-blue gradient dot represent -log10 (*P*- value)

To identify the genes and signaling pathways related to *E1*-mediated leaf development, we performed RNA-seq analysis and analyzed the differentially expressed genes (DEGs) in the *E1*-*OE* transgenic and DN50 plants. The gene expression levels were similar between two biological replicates (Fig. 3a), but differed significantly between the *E1*-*OE* transgenic lines and DN50 plants. Genes involved in metabolic process, cellular process, single-organism process, response to stimulus, and biological regulation were enriched in the DEGs (Fig. 3b). We compared the RNA- seq datasets and identified a total of 7407 DEGs (FDR *P* < 0.01) (Additional file 2: Data S1). Among these, 3966 genes were significantly upregulated and 3441 genes were significantly downregulated (Fig. 3c). The Kyoto Encyclopedia of Genes and Genomes (KEGG) pathway analysis demonstrated that some primary metabolic pathways that are essential for plant growth and development were significantly enriched; these included fatty acid metabolism, phenylpropanoid biosynthesis, cysteine and methionine metabolism, and starch and sucrose metabolism (Fig. 3d).

**Fig. 4:**
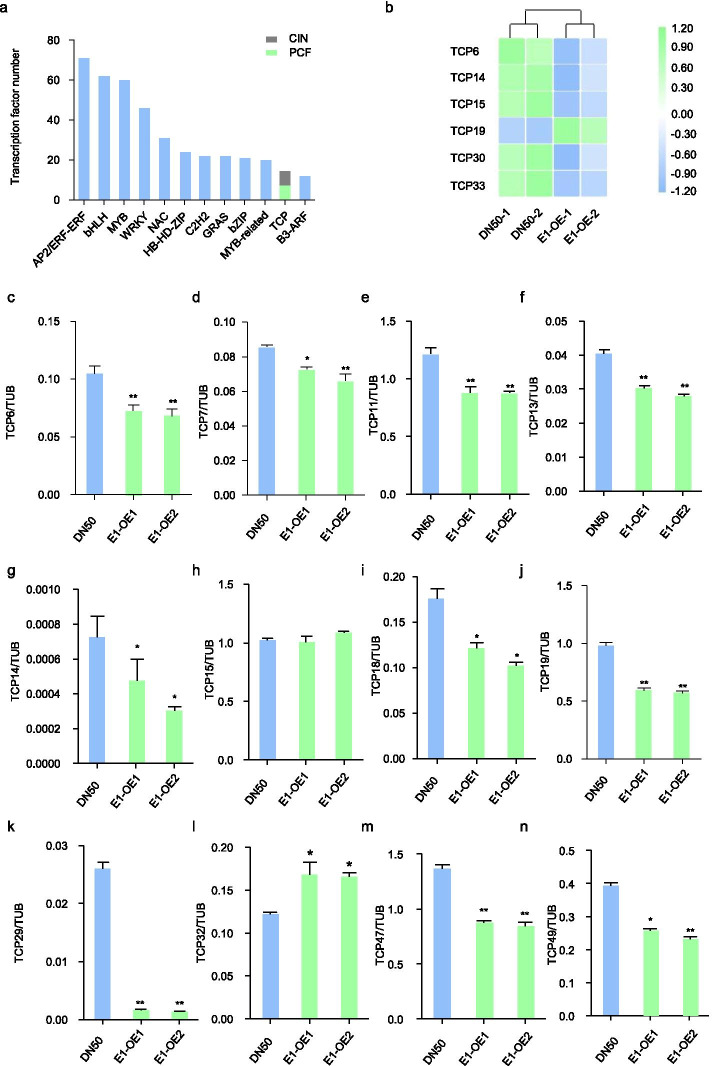
E1 negatively regulates the expression of *TCP* family genes in soybean. **a**. Numbers of transcription factors among differentially expressed genes in DN50 and *E1-OE* lines based on RNA-seq data. Green represents PCF subclass *TCP* genes, grey represents CIN subclass *TCP* genes. **b**. The heat map of differential expression of CIN subclass *TCP* genes in DN50 and *E1-OE* plants. The numerical values for the blue-to-green gradient bar represent log2- (fold change) relative to the control sample. **c**-**n**. The transcription levels of *TCP5*, *TCP7*, *TCP11*, *TCP13*, *TCP14*, *TCP15*, *TCP18*, *TCP19*, *TCP29*, *TCP32*, *TCP47* and *TCP49* in DN50 and *E1-OE* plants; data obtained by qRT-PCR. Significant differences were analyzed based on the results of three biological replicates, each with three technical replicates (Student’s *t*-test: *P < 0.05, **P < 0.01). Bars indicate the standard error of the mean

RNA-seq approaches have identified transcription factor (TF) gene families, such as the *AP2*/*ERF-ERF*, *bHLH*, *MYB*, *WRKY*, *NAC*, *HB-HD-ZIP*, *C2H2*, *GRAS*, *bZIP*, *MYB*-related, *TCP* and *B3-ARF* families (Fig. 4a). Many studies suggested that the CIN subclass TCP genes, played the important role in regulating the leaf development [31–33]. We found 14 *TCP* TF genes among the DEGs, of which six were CIN-type *TCP* genes (Fig. 4a). The heat map showed that 5 CIN-type *TCP* (*TCP6*, *TCP14*, *TCP15*, *TCP30*, and *TCP33*) genes were significantly downregulated in *E1-OE* transgenic lines as compared with DN50 (Fig. 4b).

### E1 represses *TCP* genes

The soybean genome includes 19 CIN-subclass *TCP* genes [30]. To validate the RNA-seq results and E1 regulation in all 19 CIN-type *TCP* genes in soybean, we tested their expression by qRT-PCR in *E1-OE* and DN50 plants. Most genes were downregulated in *E1-OE* transgenic plants, including *TCP6*, *TCP7*, *TCP11*, *TCP13*, *TCP14*, *TCP18*, *TCP19*, *TCP29*, *TCP47*, and *TCP49* (Fig. 4c-n). However, the expression levels of *TCP15*, *TCP36*, *TCP39*, and *TCP42* remained unchanged (Additional file 1: Fig. S3b, d, e), and *TCP32*, *TCP33* and *TCP37* were upregulated in *E1-OE* transgenic (Additional file 1: Fig. S3a, c). The expression levels of *TCP30* and *TCP38* were not detected in *E1-OE* and DN50 plants.

**Fig. 5:**
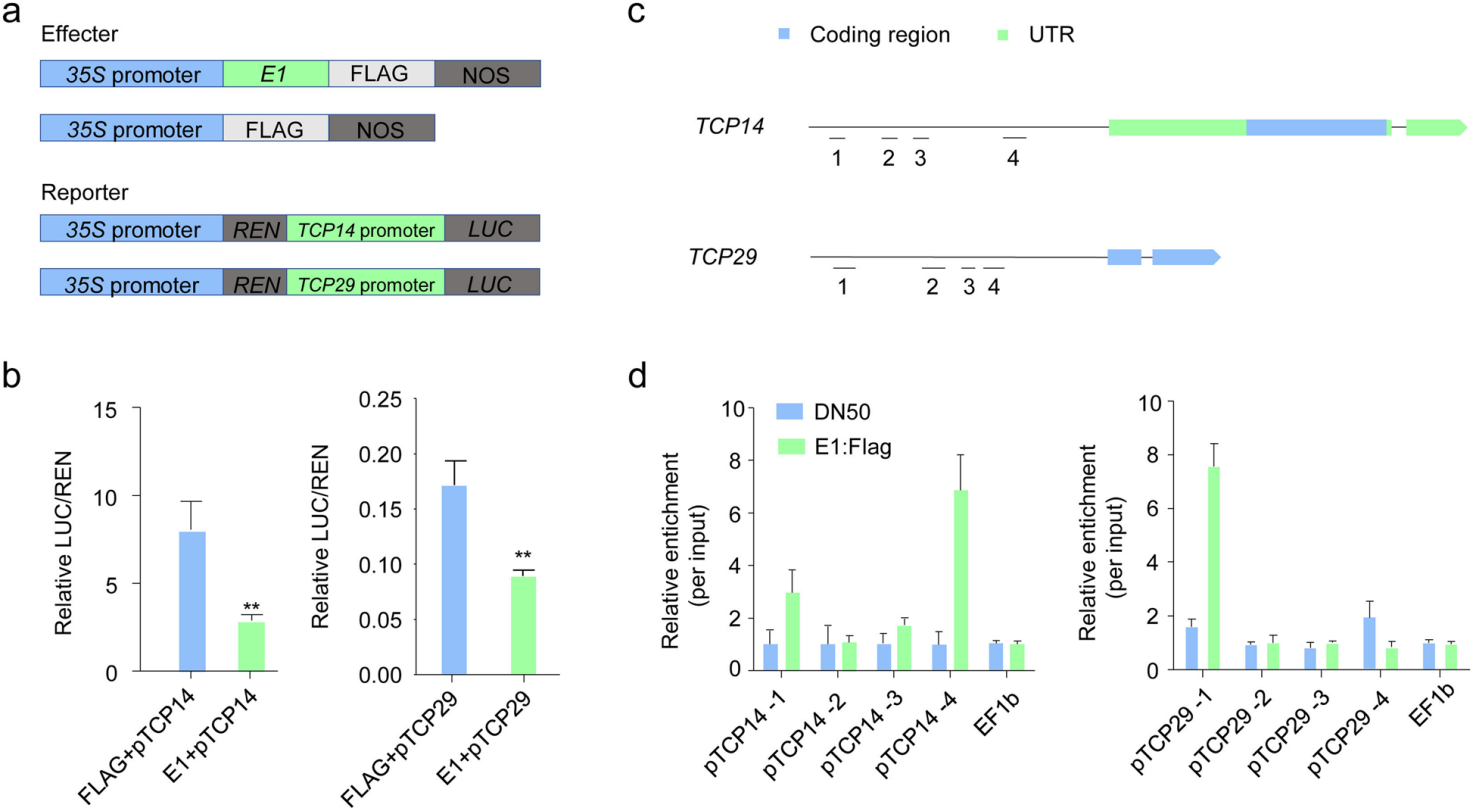
E1 represses *TCPs* transcription by directly binding to the promoter. **a**. Constructs used for the transient transfection assay. **b**. Luciferase activity under the control of *TCP14 and TCP29* promoters, from three biological replicates. A Student’s *t*-test was used to detect statistically significant differences. **c**. Location of the promoters of *TCP14* and *TCP29* and the amplicons targeted in ChIP-qPCR assay. **d**. Results of ChIP-qPCR on *TCP* amplicons in DN50 and *E1*-*OE* lines fused with Flag tags. A monoclonal Flag antibody was used for the ChIP assay

To examine the regulatory effect of E1 on its target genes, we performed transient expression assays, using *TCP14* and *TCP29* promoters fused to the *LUC* reporter (*pTCP14:LUC* and *pTCP29:LUC*; Fig. 5a). The effector construct harbored *E1* under the control of the 35S promoter (*p35S:E1*; Fig. 5a). We transformed the reporter construct (*pTCP14:LUC* or *pTCP29:LUC*) and the effector construct (*p35S:E1*) into healthy *N. benthamiana* leaves and found that E1 significantly repressed *TCP14* and *TCP29* expression (Fig. 5b). Thus, E1 regulates leaf development by repressing CIN-type *TCPs*.

To determine whether E1 directly inhibits the expression of *TCP* genes, we performed a ChIP-qPCR assay to compare the relative enrichment of specific *TCP14* and *TCP29* sequences in *E1*-*OE* and DN50 plants using anti-Flag antibodies. We selected four sites in the 2027 bp and 2209 bp regions upstream of the *TCP14* and *TCP29* promoters, respectively (Fig. 5c). The E1 protein was highly enriched in the *TCP14* promoter sites 1 and 4, and in the TCP29 promoter site 1, whereas it was present at extremely low levels in the DN50 control (Fig. 5c). These results showed that E1 could directly bound the promoters of *TCP* genes.

### The transcript levels of the CIN-type *TCP* genes in soybean tissues

**Fig. 6:**
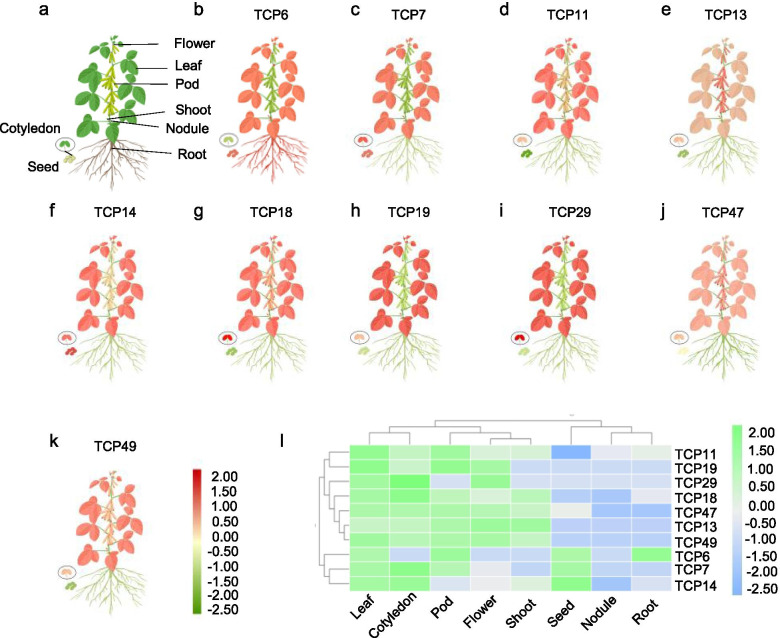
The differential expression of representative CIN-type *TCP* genes in different tissues. **a**. A model of the soybean plant. **b**-**k**. The expression of *TCP6*, *TCP7*, *TCP11*, *TCP13*, *TCP14*, *TCP18*, *TCP19*, *TCP29*, *TCP47*, and *TCP49* in different soybean tissues (flower, leaf, pod, shoot, nodule, cotyledon, seed and root) based on an RNA-seq database. The mean transcription values were visualized by TBtools; red represents high transcript level and green represents low transcript level. l. Expression levels of *TCP6*, *TCP7*, *TCP11*, *TCP13*, *TCP14*, *TCP18*, *TCP19*, *TCP29*, *TCP47*, and *TCP49* in different soybean tissues (flower, leaf, pod, shoot, nodule, cotyledon, seed and root) by qRT-PCR. The mean transcription values were visualized by TBtools; green represents high transcript levels and blue represents low transcript levels. The mean expression value was calculated from three independent biological replicates

To understand the functions of CIN-type *TCP* genes in soybean, we use an RNA-seq database and retrieved transcript levels of 10 of the *TCP* genes repressed by E1, in eight different tissues (flower, leaf, pod, shoot, nodule, cotyledon, seed and root; Machado et al., 2020). These genes presented similar expression profiles and were constitutively expressed to high levels in the leaf and cotyledon (Fig. 6a-k). In contrast, all CIN-type *TCP* genes displayed low transcript abundance in nodule and root, except *TCP6* high expression level in seed and root (Fig. 6a-k). Moreover, *TCP13*, *TCP47*, and *TCP49* presented similar expression profiles and were highly expressed in pod, flower and shoot (Fig. 6b, e, j, k). *TCP7*, *TCP14*, *TCP19*, *TCP11*, *TCP18*, and *TCP29* were expressed in shoot and seed, seed, flower and shoot, shoot, pod and shoot, and flower, respectively (Fig. 6c, f, h, d, g, i).

To determine the tissue-specific expression patterns of *TCP* genes*,* we assayed the transcript levels of 10 CIN-type *TCP* genes by qRT-PCR. The tissue-specific expression patterns in the qRT-PCR were similar to those in the RNA-seq data (Fig. 6l). Thus, the CIN-type *TCP* genes regulated by E1 play key roles in soybean leaf development.

## Discussion

The role of *E1* in controlling soybean flowering time and maturity is well known; the molecular mechanisms have also been reported [19, 21, 22, 35]. When *E1* is knocked out in soybean, flowering is promoted by the derepression of two important *FT* genes (*FT2a* and *FT5a*) under long-day conditions [19, 35]. To explore the functions of E1 in other development pathways in soybean, we created *E1*-*OE* transgenics and compared them with the DN50 wild type plants. *E1*-*OE* transgenic lines flowered significantly later under long-day conditions (Additional file 1: Fig. S1b). Consistent with previous reports [19, 35], our findings demonstrate that *E1* functions as a flowering repressor in soybean. We observed smaller, lighter, and curlier unifoliolate leaves in *E1*-*OE* transgenic plants (Fig. 1, Additional file 1: Fig. S1), suggesting that *E1* might play an important role in leaf development.

The size and shape of a leaf are major traits that affect yield in soybean [36–40]. The narrow leaf trait is associated with increased seed number than broad leaf trait in soybean [36]. The locus that controls leaf shape cosegregates with the locus that controls the seed number [40]. For example, Jeong et al. [40] recorded the numbers of 1-, 2-, 3-, and 4-seeded pods and leaf shape for each of the soybean plants, and found that narrow leaf linked with 4-seeded pods. Only one gene for leaf shape has been identified by map-based cloning in soybean —an allele of *ln* on chromosome 20, encoded by *JAGGED1* (*Glyma20g25000.1*), the ortholog of *Arabidopsis JAGGED* (*JAG*) [41]. *JAG1* complements leaf shape and silique length in *Arabidopsis* mutants [41]. Although leaf traits are important for soybean yield, the molecular link between the two remains unknown. In this study, we show that overexpression of *E1* in transgenic plants could affect unifoliolate leaf development and plant development (Fig. 1c-e, Additional file 1: Fig.S1a). Our data thus provide valuable information about the molecular basis of leaf development in soybean.

In plants, cell number and size affect leaf morphology [42–45]. In rice, the *narrow leaf 7* (*nal7*) mutant has larger but fewer bulliform cells than the wild type (Haymasari), resulting in reduced leaf width [44]. The loss of function of *ADL1* (*Adaxialized leaf 1*) increases the number of bulliform cells, which leads to a change in leaf shape in rice [42]. Our data showed that cells were more disorganized in *E1*-*OE* plants; the numbers of bulliform cells, palisade tissue cells, and spongy tissue cells were significantly higher (Fig. 2a, c-e). Thus, we propose that cell number affects the shape of the leaf. However, the molecular mechanisms underlying this effect require further study in soybean.

*E1*, a potential B3-like transcription factor, may directly target downstream genes involved in leaf development. We performed RNA-seq and found a significant enrichment in *E1*-*OE* transgenic soybean plants of metabolic pathways essential for plant growth and development (Fig. 3d). Therefore, E1 may regulate leaf development through metabolic signaling pathways in soybean. Furthermore, Furthermore, among the TF families previously identified by RNA-seq, such as the *AP2*/*ERF-ERF*, *bHLH*, *MYB*, *WRKY*, *NAC*, *HB-HD-ZIP*, *C2H2*, *GRAS*, *bZIP*, *MYB*-related, *TCP* and *B3*-*ARF* TFs (Fig. 4a). CIN-type *TCP* genes in particular appear to regulate leaf development [9, 12, 13]. For example, CIN-like TCP proteins promote leaf differentiation by regulating the meristematic and auxin response genes in *Arabidopsis* [46]. In soybean, we found that most CIN-type *TCP* genes were down-regulated in *E1*-*OE* lines. E1 also repressed *TCP14* and *TCP29* expression in transient expression assay, and our ChIP-qPCR analysis demonstrated that E1 directly binds to the *TCP14* and *TCP29* promoter (Fig. 5d). Therefore, E1 may directly repress CIN-type *TCP* genes to regulate the leaf development in soybean.

## Conclusions

Based on our data, we determined that the overexpression of *E1* could affect leaf development in soybean by directly repressing a large number of leaf development-related CIN-type *TCP* genes. E1, therefore, regulates leaf development and flowering time. Our findings provide important information into the molecular mechanism underlying leaf development in soybean.

## Methods

### Plasmid construction and soybean transformation

For overexpression and *EI-Flag* fusion constructs, we amplified the coding sequence (CDS) of *E1* using the primer set *E1Flag-F*/*E1Flag-R*. The amplicon was inserted into PTF101-*3Flag* under the control of a CaMV35S promoter. The PCR conditions were as follows: 94 °C for 2 min followed by 30 cycles at 94 °C for30s, 55 °C for 30s, and 72 °C for 30s and a final extension at 72 °C for 10 min. Cotyledonary nodes from DN50 and W82 were used as explants for the *Agrobacterium tumefaciens*-mediated transformation method described by Paz et al. [47]. Transgenic soybean plants (T_1_) were identified by PCR amplification and western blot hybridization, and then were advanced to T_7_ for further analysis. All primers used for vector construction, PCR, and qRT-PCR assays for target genes are listed in Additional file 4: Table S1.

### Materials and growth conditions

DN50 and W82, were obtained from the Innovative Center of Molecular Genetics and Evolution, School of Life Sciences, Guangzhou University, Guangzhou, and subsequently used for transformation and experiments. The *E1*-*OE* transgenics and wild types (W82, DN50) were grown in a chamber maintained at 25 °C and 70% relative humidity with a 16 h light/ 8 h dark cycle. Plants were phenotyped 7 days after emergence (DAE).

*Nicotiana benthamiana* was obtained from the Innovative Center of Molecular Genetics and Evolution, School of Life Sciences, Guangzhou University, Guangzhou, and used for transactivation assays. The *N.benthamiana* seeds were grown in a chamber maintained at 22 °C and 70% relative humidity with a 12 h light/ 12 h dark cycle. Twenty days after planting, the leaves were used for transient transformation.

### Quantitative reverse transcription-PCR (qRT-PCR) analysis

qRT-PCR analysis was performed to determine the transcript abundance of *E1*. Total RNA was isolated from DN50 soybean leaf, cotyledon, pod, flower, shoot, seed, nodule and root using TRIzol (Invitrogen, Shanghai, China) according to the manufacturer’s protocol. cDNA was synthesized using Oligo (dT) 18 primer and the First cDNA transcriptase kit (Takara, Dalian, China). qRT-PCR was performed using a LightCycler 480 SYBR Green I Master (Roche, Mannheim, Germany) in Roche LightCycler480 system (Roche, Mannheim, Germany). The soybean housekeeping gene *Tubulin* was used as the internal control. The relative transcript level of the target gene was calculated using the 2^−ΔΔCt^ method. Three biological replicates with three technical replicates each were performed.

### RNA-seq analysis

Two independent *EI-OE* transgenic lines and two DN50 plants grown for 20 days in the greenhouse were used for RNA-sequencing (RNA-seq) analysis. Total RNA was extracted from leaves using the Spectrum Plant Total RNA Kit (Sigma-Aldrich). The RNA was sequenced on an Illumina HiSeq 2500 platform to generate paired-end reads. DEGs between samples were defined by DESeq using two separate models [48], based on fold change > 2 and false discovery rate–adjusted *P* value < 0.05. DEGs were then subjected to enrichment analysis of Gene Ontology (GO) functions and Kyoto Encyclopedia of Genes and Genomes (KEGG) pathways.

### Transient expression assay

A transient dual-luciferase assay was performed as previously described [49]. Briefly, *p35S:E1-Flag* was used in effector constructs and the 2027 bp and 2209 bp promoter sequences of *TCP14* and *TCP29* were cloned using gene-specific primers *pTCP14/29luc*-F/R and inserted into the pGreen-0800-LUC vector. The reporter construct *pTCPs:LUC* and the effector constructs *p35S:E1-Flag* were transformed into *A. tumefaciens* strain GV3101 and transfected into healthy leaves of 21-d-old *N. benthamiana* tobacco leaves by agroinfiltration as described previously [26, 50]. The plants were placed under continuous white light for 3 d after infiltration, leaf samples were collected for the Dual-Luciferase Reporter Assay System kit (Promega, USA). Relative LUC activity was normalized against the renilla luciferase (REN) activity, and the data presented are the averages of at least three independent replicates.

### Protein extraction and immunoblotting

To analyze protein expression in transgenics, total proteins were extracted according to the protocol of Cheng et al. [26]. Total proteins were transferred to polyvinylidene difluoride membranes (Millipore, Germany) and probed using anti-Flag antibodies (Sigma).

### Chromatin immunoprecipitation–qPCR assays

For chromatin immunoprecipitation (ChIP)–qPCR assays, DN50 and *E1-OE* transgenic lines were subjected to chromatin extraction and immunoprecipitation as described by Saleh et al. [51]. The precipitated DNA was recovered and analyzed by qRT-PCR with LightCycler 480 SYBR Green I Master (Roche, Mannheim, Germany). The precipitated input DNA samples were analyzed by qPCR using gene-specific primers. The data were normalized to input transcript levels and the means represent three biological replicates.

### Tissues expression profile analysis

The expression data for *TCP* genes in different tissues, including leaf, shoot, root, flower, seed, pod, cotyledon, and nodule, were available in the RNA-seq database [52]. TBtools [53] was used to display the expression profile of *TCP* genes in the heatmap.

## Supplementary Information

**Additional file 1: Fig. S1.** Phenotypes of *E1* transgenic lines and DN50 plants under LD (long day, 16 h light/8 h dark) conditions. **Fig. S2.** The leaf phenotypic characterization of the E1-overexpression (*E1-OE*) plants in W82. **Fig. S3.** The transcription levels of *TCP33*, *TCP36*, *TCP37*, *TCP39* and *TCP42* in DN50 and *E1-OE* plants.

**Additional file 2: Fig. S4.** Source data of full gel blot images for Fig.1a.

**Additional file 3: Data S1.** List of genes with significant expression changes. (XLS 6546 kb)

**Additional file 4: Table S1.** Primers used for PCR and qRT-PCR in this study.

## Data Availability

The datasets and materials developed and analyzed in this study are available from the corresponding author on reasonable request. The RNA-seq data that support the findings of this study have been deposited in to NCBI with accession number PRJNA749473 (https://www.ncbi.nlm.nih.gov/bioproject/PRJNA749473).

## Abbreviations

E1-OE: E1-overexpression
DN50: DongNong 50
W82: Williams 82
CIN: CINCINNATA
TCP: TEOSINTE BRANCHED1/CYCLOIDEA/PROLIFERATING CELL FACTOR
WT: Wild type
LD: Long-day
JAW: JAGGED AND WAVY
CYC/TB1: CYCLOIDEA/ TEOSINTE BRANCHED1
CTR: Cell tense ratio
SR: Spongy tissue ratio
DEGs: Differentially expressed genes
TF: Transcription factor
nal7: Narrow leaf 7
ADL1: Adaxialized leaf 1
CDS: Coding sequence
qRT-PCR: Quantitative reverse transcription-PCR
RNA-seq: RNA-sequencing
GO: Gene Ontology
KEGG: Kyoto Encyclopedia of Genes and Genomes
REN: Renilla luciferase
ChIP: Chromatin immunoprecipitation
