# Supplementary material for: The legume-specific transcription factor E1 controls leaf morphology in soybean
Source: BMC Plant Biol. 2021 Nov 13;21:531. doi: 10.1186/s12870-021-03301-1 (PMC8590347; doi:10.1186/s12870-021-03301-1)
Supplement: Supplementary file 1 — Additional file 1: Fig. S1. Phenotypes of E1 transgenic lines and DN50 plants under LD (long day, 16 h light/8 h dark) conditions. Fig. S2. The leaf phenotypic characterization of the E1-overexpression (E1-OE) plants in W82. Fig. S3. The transcription levels of TCP33, TCP36, TCP37, TCP39 and TCP42 in DN50 and E1-OE plants. [file 12870_2021_3301_MOESM1_ESM.pdf]

Additional file 1:

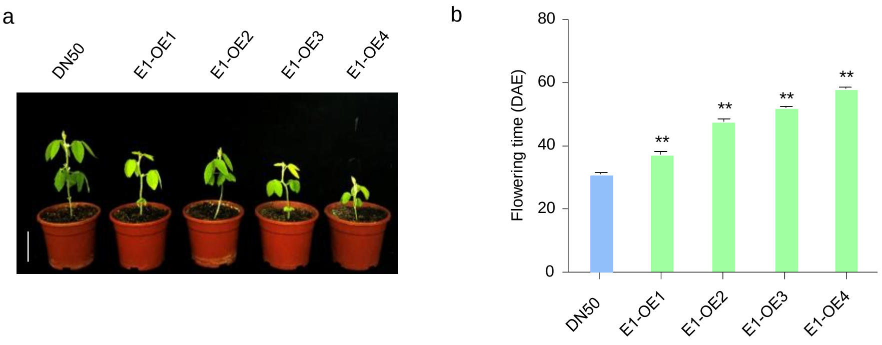

Fig. S1. Phenotypes of *E1-OE* transgenic and DN50 plants under LD (long day, 16 h light/8 h dark) conditions.
a. Comparison of DN50 and *E1-OE* transgenic seedings. b. Flowering time. Flowering time was recorded at the R1 stage (days from emergence to the appearance of the first open flower in 50% of the plants). DAE, days after emergence.

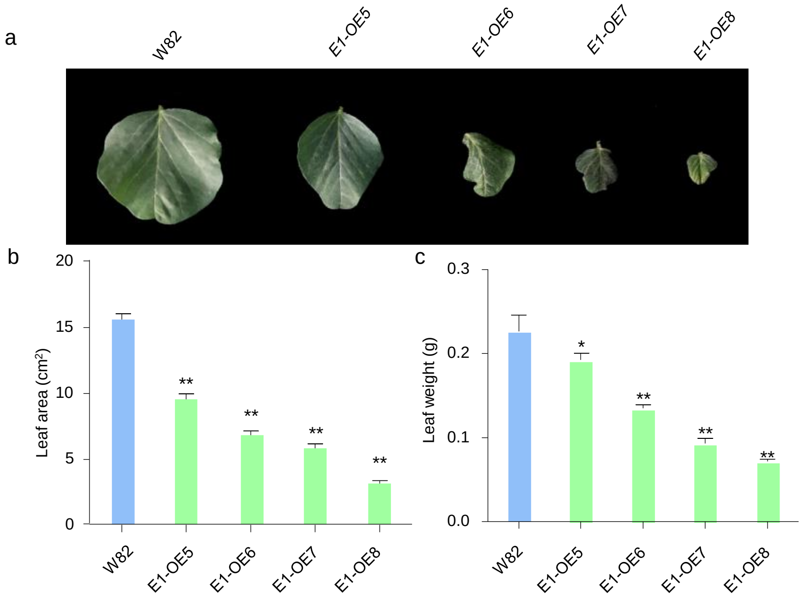

Fig. S2. The leaf phenotypic characterization of the *E1-overexpression* (*E1-OE*) plants in W82. a. Top view of W82 and *E1-OE* plants. b. Quantification of leaf size in W82 and *E1-OE* plants. c. Weight of leaf size in W82 and *E1-OE* plants. All values are presented as mean ± standard error of the mean (s.e.m.). (n = 13 plants). Bars indicate the s.e.m. Significant differences were identified by Student's t-test (***P* < 0.01).

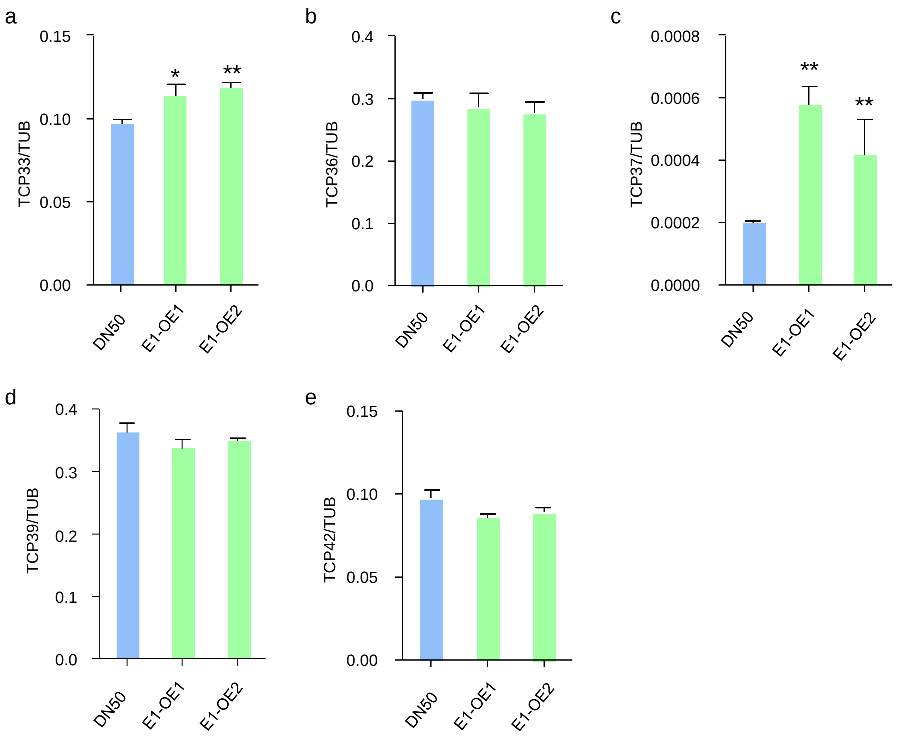

Fig. S3. The transcription levels of *TCP33*, *TCP36*, *TCP37*, *TCP39* and *TCP42* in DN50 and *E1-OE* plants.

a. The expression of *TCP33*. b. The expression of *TCP36*. c. The expression of *TCP37*. d. The expression of *TCP39*. e. The expression of *TCP42.* Values shown are relative to the control gene TUB and represent means ± s.e.m. of three biological replicates with three technical replicates. Significant differences were identified by Student's t-test (**P* < 0.05 , ***P* < 0.01).
