# Supplementary material for: The legume-specific transcription factor E1 controls leaf morphology in soybean
Source: BMC Plant Biol. 2021 Nov 13;21:531. doi: 10.1186/s12870-021-03301-1 (PMC8590347; doi:10.1186/s12870-021-03301-1)
Supplement: Supplementary file 2 — Additional file 2: Fig. S4. Source data of full gel blot images for Fig.1a. [file 12870_2021_3301_MOESM2_ESM.pdf]

Additional file 2:

a

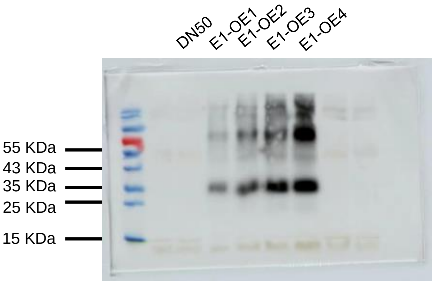

b

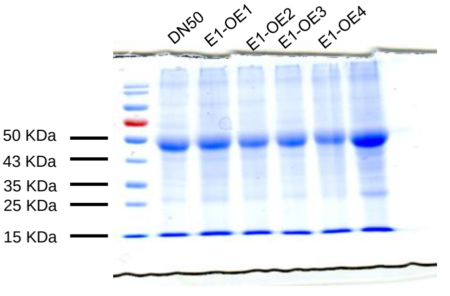

Figure S4. Source data of full gel blot images for figure 1a. a, original gel blot image of figure 1a, upper panel. b, original image of figure 1a, lower panel.
