# Supplementary material for: The legume-specific transcription factor E1 controls leaf morphology in soybean
Source: BMC Plant Biol. 2021 Nov 13;21:531. doi: 10.1186/s12870-021-03301-1 (PMC8590347; doi:10.1186/s12870-021-03301-1)
Supplement: Supplementary file 4 — Additional file 4: Table S1. Primers used for PCR and qRT-PCR in this study. [file 12870_2021_3301_MOESM4_ESM.doc]

Table S1 Primers used for PCR and qRT-PCR in this study.

Primers for constructs and sequencing

| Primers name | Primers sequences (5’-3’) |
| --- | --- |
| pTCP14-2027-F | CAAAGGGCATCCCGACAA |
| pTCP14-2027-R | TTTTCTTGCATGTTCGGATG |
| pTCP29-2209-F | TGGGTGTCGTGTATAGTTT |
| pTCP29-2209-R | GCCCCTTGTGATTGTTCC |
| pTCP14-2027-lucF | GTCGACGGTATCGATAAGCTTCAAAGGGCATCCCGACAAACACATT |
| pTCP14-2027-lucR | CGCTCTAGAACTAGTGGATCCTTTTCTTGCATGTTCGGATGAAGCA |
| pTCP29-2209-lucF | GTCGACGGTATCGATAAGCTTTGGGTGTCGTGTATAGTTTGGAAT |
| pTCP29-2209-lucR | CGCTCTAGAACTAGTGGATCCGCCCCTTGTGATTGTTCCTCCTC |
| p35S-E1F | GAGAACACGGGGGACTCTAGAATGAGCAACCCTTCAGATGA |
| p35S-E1R | ATCCTTGTAGTCCATGGATCCATTCTCTGGCATAGCTTG |

Primers for ChIP-qPCR

| Primers name | Primers sequences (5’-3’) |
| --- | --- |
| PYTCP14-1 F | TAATATGGTAATACAAATTTGGCTT |
| PYTCP14-1 R | TGTATCCATCCAACTTCTGCTT |
| PTCP14-2 F | TATCTCTAAATAAATGTTTTTCAGT |
| PTCP14-2 R | GTTATGTCAATATTTACTAGCAGAG |
| PTCP14-3 F | GAGAAAGAGAAGAGTGAATGGGT |
| PTCP14-3 R | CCACACTACACTACACTACACACAA |
| PTCP14-4 F | ACAACCCATTAGGAATAAGTGC |
| PTCP14-4 R | TCTTGGTCTTCTTGGTTTTCTC |
| PTCP29-1 F | ATTTTGTTTTGGGAATCACG |
| PTCP29-1 R | CTTCGGGACAAACCAAAAT |
| PTCP29-2 F | GGGTTGTTACTGTTACCAAAGAG |
| PTCP29-2 R | CCGAGAGTCTAAAAACTAATCCT |
| PTCP29-3 F | CTTCTCTGACCTGACCCTGC |
| PTCP29-3 R | GATGGACAGAGAGTGAGGGG |
| PTCP29-4 F | TGTGCTTTTACCCTCCTTGT |
| PTCP29-4 R | AATCTTATCTATCTGCTCTTCATCT |

Primers for qRT-PCR

| Primers name | Primers sequences (5’-3’) |
| --- | --- |
| qTCP6-F | AACAGTGCTGCCTTGTATAGAG |
| qTCP6-R | CATATTCCCACCAATCCCAGAA |
| qTCP7-F | AACAGTGCTGCCTTGTATAGAG |
| qTCP7-R | CATATTCCCACCAATCCCAGAA |
| qTCP11-F | AACAGTGCTGCCTTGTATAGAG |
| qTCP11-R | CATATTCCCACCAATCCCAGAA |
| qTCP13-F | AACAGTGCTGCCTTGTATAGAG |
| qTCP13-R | CATATTCCCACCAATCCCAGAA |
| qTCP14-F | AACAGTGCTGCCTTGTATAGAG |
| qTCP14-R | CATATTCCCACCAATCCCAGAA |
| qTCP15-F | AACAGTGCTGCCTTGTATAGAG |
| qTCP15-R | CATATTCCCACCAATCCCAGAA |
| qTCP18-F | AACAGTGCTGCCTTGTATAGAG |
| qTCP18-R | CATATTCCCACCAATCCCAGAA |
| qTCP19-F | AACAGTGCTGCCTTGTATAGAG |
| qTCP19-R | CATATTCCCACCAATCCCAGAA |
| qTCP29-F | AACAGTGCTGCCTTGTATAGAG |
| qTCP29-R | CATATTCCCACCAATCCCAGAA |
| qTCP30-F | AACAGTGCTGCCTTGTATAGAG |
| qTCP30-R | CATATTCCCACCAATCCCAGAA |
| qTCP32-F | AACAGTGCTGCCTTGTATAGAG |
| qTCP32-R | CATATTCCCACCAATCCCAGAA |
| qTCP33-F | AACAGTGCTGCCTTGTATAGAG |
| qTCP33-R | CATATTCCCACCAATCCCAGAA |
| qTCP36-F | AACAGTGCTGCCTTGTATAGAG |
| qTCP36-R | CATATTCCCACCAATCCCAGAA |
| qTCP37-F | AACAGTGCTGCCTTGTATAGAG |
| qTCP37-R | CATATTCCCACCAATCCCAGAA |
| qTCP38-F | AACAGTGCTGCCTTGTATAGAG |
| qTCP38-R | CATATTCCCACCAATCCCAGAA |
| qTCP39-F | AACAGTGCTGCCTTGTATAGAG |
| qTCP39-R | CATATTCCCACCAATCCCAGAA |
| qTCP42-F | AACAGTGCTGCCTTGTATAGAG |
| qTCP42-R | CATATTCCCACCAATCCCAGAA |
| qTCP47-F | AACAGTGCTGCCTTGTATAGAG |
| qTCP47-R | CATATTCCCACCAATCCCAGAA |
| qTCP49-F | AACAGTGCTGCCTTGTATAGAG |
| qTCP49-R | CATATTCCCACCAATCCCAGAA |
